# Factors in the management of feeding in nursery school children as perceived by their mothers in rural Bondo County, Kenya

**DOI:** 10.1186/1472-698X-13-47

**Published:** 2013-11-15

**Authors:** Allan R Were, Felix N Kioli, Kennedy Onkware, Elizabeth O Onyango, Sussy Gumo, Collins Ouma

**Affiliations:** 1 Department of Sociology and Anthropology, Maseno University, Maseno, Kenya; 2 Centre for Disaster Management and Humanitarian Assistance, Masinde Muliro University of Science & Technology, Kakamega, Kenya; 3 Department of Public Health, Maseno University, Maseno, Kenya; 4 Department of Religion, Theology and Philosophy, Maseno University, Maseno, Kenya; 5 Department of Biomedical Sciences and Technology, Maseno University, Maseno, Kenya

**Keywords:** Feeding practices, Children, Mothers, Rural Bondo

## Abstract

**Background:**

The effects of malnutrition on health status and survival of children has been the subject of extensive research for several decades. Malnutrition affects physical growth, cognitive development of children, morbidity and mortality. The current study was an exploratory survey that focused on factors affecting feeding of nursery school children as perceived by their mothers in a rural setting in Usigu Division of Bondo County, Kenya.

**Methods:**

The sampling frame was mothers whose children were in Kanyibok, Sanda and Usenge nursery schools. Purposive sampling methods were used to draw a total of 108 respondents. In a logistic regression model, bad management of feeding was the dependent variable while factors perceived to affect management of feeding were the independent variables.

**Results:**

Married mothers were more likely to manage good feeding practices (OR, 0.34, 95% CI, 0.21-0.76; *P* = 0.022) relative to those who were single or widowed. Additional analyses showed that low education levels (OR, 7.33, 95% CI, 3.37-12.91; *P* = 0.023), younger mothers (OR, 6.04, 95% CI, 3.22-9.68; *P* = 0.029) and mothers engaged in business (OR, 4.02, 95% CI, 2.11-7.85; *P* = 0.027) increased their likelihood of not feeding the pre-school children. Majority of the children who ate the main meals in other houses belonged to young mothers in the age category of 15–29 years. Further analyses demonstrated that if the order of serving food was to the children first, then they had high likelihood of having good feeding relative to when the father was served first (OR, 0.22, 95% CI, 0.14-0.61; *P* = 0.011).

**Conclusions:**

Based on these findings, there is an urgent need for sensitization of the mothers on the management of feeding of these pre-school children in Bondo County. It is hoped that relevant interventions would then be designed with the view of managing children feeding in such rural settings as in Bondo County in Kenya.

## Background

The effects of malnutrition on human performance, health and survival have been the subject of extensive investigations for several decades. Studies have shown that malnutrition affects physical growth, morbidity, mortality and cognitive development [1]. The problems associated with food deficiency among pre-school children have become a matter of worldwide concern, especially in developing countries where there are numerous challenges [2]. Factors that contribute to malnutrition among pre-school children are as numerous as they are diverse [3]. The primary determinants of malnutrition, as conceptualized by several authors relate to unsatisfactory food intake, severe and repeated infections, or a combination of the two [4]. The United Nations reports that there is a wide variation in food consumption patterns due to dietary traditions in Kenya [5]. As a result, children have inadequate nourishment in their diets and therefore consume foods that are deficient in essential nutrients [6]. Feeding amongst pre-school children is to a large extent determined by social and economic activities of the mothers. One challenge of children in this age group is that they often have the lowest priority at meal time and therefore miss out in terms of getting required nutrients [7]. Although mothers may be aware of their children’s feeding needs, most rural women in Kenya are unable to feed their children properly because of social, economic and environmental factors.

In rural Kenya, children’s feeding is an outcome of various interacting factors such as cultural, economic, religious, agricultural as well as dietary habits [8]. According to a previous study, the extent of illiteracy in a society reflects not only on the limitations in the capacity to organize and mobilize resources, but also on the degree of social and political maturity [9]. An alternative argument to this is that education acts as a proxy for command over such resources as food [10]. It has been argued by other authors that the primary cause of nutritional problems are poor feeding, especially at the time of weaning, up to the age of five years in children [11]. In certain traditional communities, studies investigating factors affecting feeding are usually focused on particular age groups, especially on children below five years of age [12].

The Kenyan National Council for Population and Development’s estimates of the prevalence of malnutrition in 1997–1998 in children less than five years of age showed a large variation by province, reflecting the considerable variability in environmental and socio-economic risk factors [13]. These estimates were approximately 33.0% (stunting), 22.1% (underweight), and 6.1% (wasting) for Nyanza Province in western Kenya [14]. It was observed that some of the practices in these studies were linked to groups of people with a necessity of specific types of food. In many cases, nutritious foods were withheld from children for fear of adverse consequences [4,15]. Despite the various studies investigating the malnutrition in children within the country, no studies have been carried out to investigate factors affecting the management of feeding among mothers of nursery school children. Therefore, the main objective of the current study was to describe factors that affect the management of feeding in nursery school children from the mother’s perspective in Usigu Division of Bondo County, Nyanza Province, Kenya. The results would help in the formulation of appropriate policies aimed at addressing these factors in improvement of feeding among children in Kenya.

## Methods

### Study site

This study was conducted in Usigu Division of Bondo County, Nyanza Province in Kenya. The district is mainly inhabited by the Luo ethnic group. According to the Bondo District Development Plan (1994–1996), Usigu Division has an area of about 187 sq. km. with an annual rainfall of 864 mm. The district experiences an equatorial climate with strong influence from the expansive Lake Victoria. Long rains are received from March to May with April and May being the peak months. Cotton is the main cash crop in this area. Sorghum, cassava, maize and beans are the main food crops. The total area under cultivation is about 20% of the total land area. Cattle, sheep, and poultry keeping are also part of the farming activities in the district. The constraints to farming activities include lack of pasture, livestock diseases and inadequate feeds for poultry [16]. The area is characterized by a number of beaches and consequently fishing is one of the main occupations of the inhabitants [17].

### Study design and sampling

This study was an exploratory survey using a structured questionnaire, focus group discussions (FGDs) as well as direct observations to determine the factors associated with feeding of nursery school children from the mother’s perspective. The questionnaire covered demographic information, practices associated with feeding, socio-cultural and economic factors from the mother’s perspective. Mothers of nursery school children formed the sampling frame for the study. The study sample was drawn from three nursery schools namely Kanyibok (n = 58), Sanda (n = 53) and Usenge (n = 51) in Usigu Division using purposive sampling. In these schools, children only attend classes and the meals are taken absolutely at home. A total of 162 mothers with nursery schools children in these locations were interviewed. However, one hundred and eighty (108) mothers [Kanyibok (n = 39), Sanda (n = 33) and Usenge (n = 36)] of the nursery school children qualified for inclusion in the analyses based on the completion of the questionnaires.

### Research procedure

The research was approved by the Office of the President as well as the University of Nairobi and letters of request to conduct the study were sent to the respective divisional District Officers. Written informed consent was sought from the study participants’ parents/guardians prior to the interviews. Structured interviews were conducted using pre-tested questionnaires administered to mothers of nursery school children (See Additional file 1). The questionnaire covered demographic information, feeding practices, socio-cultural and economic issues from the mother’s perspective. The FGDs and direct observation were conducted using FGDs guide and observation guide, respectively. The research assistants who were involved in the interviews were pre-trained in basic interviewing techniques and the questionnaire and guides were pre-tested for flow of questions and for validity. Data collection period lasted 100 days (between January 2012 and April, 2012).

### Data analysis

Data analyses were performed using SPSS® statistical software package version 19.0 (IBM SPSS Inc., Chicago, IL, USA). Proportions between variables (marital status, education levels, age, occupation, those served food in the first order amongst all correspondents) were determined using Pearson’s chi-square. Logistic regression analyses were used to identify perceived factors that affected feeding among mothers of nursery school children. For logistic regression analyses, reference groups were defined at an OR of 1.00. Any OR value >1 was considered 'bad’ while those <1 were considered 'good’. Bad management of feeding was the dependent variable while all other factors were the independent variables in the logistic regression models. *P*-values ≤0.05 were considered statistically significant.

## Results

### Demographic characteristics and association with children’s feeding practices

Table 1 presents the demographic characteristics and their association with children’s feeding for the 108 respondents (mothers) who participated in the study. About 81% of the mothers were married, 14% were widowed, 5% were single and 1% divorced. Most of them (83%) had attained above five years of formal education, with majority having attained five to eight years of schooling. Over 50% of the respondents were engaged in business (usually selling vegetables, grains and fish), while 36% were farmers and 14% were in salaried employment.

**Table 1:** Frequencies of the demographic characteristics of the mothers and their association with management of feeding practices in children

	**n (%)**	**OR**	**95% CI**	***P*-value**
**Marital status**				
Single	5 (4.63)	1.00		
Married	87 (80.55)	0.34	0.21 – 0.76	0.022
Widowed	15 (13.89)	1.26	0.88 – 2.35	0.061
Divorced	1 (0.93)	NA		
**Education levels**				
None	9 (8.33)	1.00		
1-4 years	10 (9.26)	7.33	3.37 – 12.91	0.023
5-8 years	56 (51.85)	5.20	2.65 – 7.89	0.031
≥9 years	33 (30.56)	3.88	1.76 – 6.55	0.039
**Age**				
18-24 years	15 (13.89)	1.00		
25-29 years	32 (29.63)	6.04	3.22 – 9.68	0.029
30-34 years	27 (25.00)	4.28	2.09 – 7.49	0.041
35-39 years	13 (12.04)	3.01	1.88 – 5.72	0.044
40-44 years	11 (10.18)	1.99	1.02 – 3.23	0.046
≥45 years	10 (9.26)	1.01	0.99 – 2.54	0.055
**Occupation**				
Salaried	15 (13.89)	1.00		
Farming	39 (36.11)	1.44	1.01 – 2.32	0.034
Business	54 (50.00)	4.02	2.11 – 7.85	0.027

Values are n (%). OR = Odd Ratio; 95% CI = 95% Confidence Interval. Logistic regression analyses were performed using the first category in each of the demographic characteristic as the reference group. Bad management of feeding was the dependent variable while all other factors were the independent variables in the logistic regression models.

Additional logistic regression analyses demonstrated that children for married mothers were more likely to have good feeding (OR, 0.34, 95% CI, 0.21-0.76; *P* = 0.022) relative to those who were single or widowed. We could not statistically compute values for the divorced, since there was only 1 respondent. Additional analyses showed that low education levels (OR, 7.33, 95% CI, 3.37-12.91; *P* = 0.023), younger mothers (OR, 6.04, 95% CI, 3.22-9.68; *P* = 0.029) and mothers engaged in business (OR, 4.02, 95% CI, 2.11-7.85; *P* = 0.027) increased the likelihood of their children not feeding (Table 1).

### Feeding habits of nursery school children

Table 2 presents the feeding habits of the nursery school children stratified as per their mothers’ age. As expected among most rural communities in Kenya, children of young mothers ate elsewhere around the homestead (*dala*). In addition, majority of the children who ate the main meals in other houses i.e. stepmothers', grandmothers’ and neighbors’ houses belonged to young mothers in the age category of 15–29.

**Table 2:** Frequencies of children eating habits as stratified by mother’s age

**Mother’s age (yrs)**	**Neighbors’**	**SM**	**GM**	**Other**	**None**	**Total**
	**n (%)**	**n (%)**	**n (%)**	**n (%)**	**n (%)**	**n (%)**
15-29	15 (40.54)	7 (43.75)	13 (61.91)	3 (33.3)	9 (36)	47 (43.52)
30-39	13 (35.13)	7 (43.75)	6 (28.57)	4 (44.45)	10 (40.00)	40 (37.04)
≥40	7 (18.92)	2 (12.05)	2 (9.52)	2 (22.22)	5 (18.00)	18 (16.66)
NS	2 (5.41)	0 (0.00)	0 (0.00)	0 (0.00)	1 (3.00)	3 (2.78)
Total	37	16	21	9	25	108

Table legend: Results are presented as n (%). SM = Stepmothers, GM = Grandmothers, NS = Not specified.

During focus group discussions, it emerged that majority of the mothers recognized the fact that it was culturally-acceptable for children to eat in other places or households within the family. One respondent said: “*Children are not only mine but they belong to the whole family and they can eat anywhere within the family. I have no control over that”.*

Another respondent said that: *“For children to be socialized well they should be allowed to eat anywhere and interact within the extended family”.*

While it is nutritionally justifiable for children to eat in other houses within the homestead, it can be argued that the tendency or habit of children to eat the main meal in other houses other than their mothers', could as well imply a lack of adequate care for the children by their young mothers in the community. Majority (61.91%) whose mothers were in the age category of 15–19 years took their main meals at their grandmother’s houses.

### Order of serving meals at the household

Table 3 presents data on the order of being served food in the households from which the mothers were drawn from. Contrary to expectations, children in this rural community were served first during the main meals. Majority of the mothers (39.81%) reported that children were usually served first during the main meal. Only 25 (23.15%) of the mothers reported that they served their husbands (the children’s fathers) first. This was confirmed by some respondents who said indicated reasons for serving the husband first as, “*Because he is the head of the house and family”.*

**Table 3:** Frequencies of the order of being served food within the household and their association with management of feeding practices in children

**Person being served**	**n (%)**	**OR**	**95% CI**	***P*-value**
Father	25 (23.15)			
Children	43 (39.81)	0.22	0.14 – 0.61	0.011
Together	36 (33.33)	0.73	0.43 – 0.92	0.019
No Response	4 (3.71)	N/A		
**Total**	**108**			

Values are n (%). OR = Odd Ratio; 95% CI = 95% Confidence Interval. Logistic regression analyses were performed using the first category in each of the 'Persons being served’ as the reference group. N/A = Not applicable. Bad management of feeding was the dependent variable while all other factors were the independent variables in the logistic regression models.

Another respondent said*: “This is my husband whom I must respect and therefore should serve him first before children”.*

Another 33% of the respondents reported that the family usually took their main meals together. Additional logistic regression analyses demonstrated that if the order of serving food was to the children first, then they had high likelihood of having good feeding relative to when the father was served first (OR, 0.22, 95% CI, 0.14-0.61; *P* = 0.011). The odds of having good feeding habits reduced as the children were served food together with all the rest of the households (OR, 0.73, 95% CI, 0.43-0.92; *P* = 0.019; Table 3).

## Discussion

The current study was designed with the main objective of describing the factors that affect feeding of nursery school children from the mother’s perspective in Usigu Division of Bondo County, Nyanza Province, Kenya. Results presented here demonstrate that lower age, low education level and the mother’s getting engaged in business negatively affected their children’s feeding. In addition, it was demonstrated that majority of mothers within the age range of 15–19 years had most of their children feeding at their grandmothers’ houses. The order in which food was served in the household also affected the eating habits of the children since if they were served first, there was a higher likelihood of them (children) having been fed well.

In the current study, it was observed that the economic activities of the mothers (particularly those who were engaged in business) affected their children’s feeding. For example, children whose mothers were in business could not get food at lunch time because it was during that time that most mothers were out at the market selling their fish. As such, they could not spare time to make food for their children. This observation agrees with the argument of a previous study [10] that showed that women’s economic activities have an impact on child care, especially if the activity is incompatible with child rearing or where the mother lacks access to another person, who may assume responsibility and take care of the child. This implies that the management of children’s feeding may be significantly affected by the nature of the mother’s employment. Regarding expectations on the mother’s level of educational attainment, it was observed that the higher the education level attained, the higher the likelihood of the mothers effectively being able to manage a good feeding for their children. This was effective despite the fact that the study was conducted in a rural setting in which cultural values related to child rearing are still in place.

In terms of feeding habits, data show that most children took their main meals elsewhere before coming to their mothers’ houses to sleep. The consequence of this was the children not taking a keen interest on taking the food at their mothers’ houses. This practice of allowing children to eat at the extended family members’ houses is usually perceived as a socialization process for the young mothers and their children. Because of the exogamous and virilocal marriage practices among the Luo people, children often grow up in the homes of their paternal grandparents where they share many experiences including food. This supports the arguments by other scholars that one of the effective ways through which grandmothers enhanced their relations with their grandchildren were through sharing food [18]. Given that young mothers in the study area spend part of their time in their mother in-laws’ houses when newly-married, they had no alternative other than allowing their children to eat at their grandmothers’ houses. This in itself could imply to the young mothers, a reduced control over which type of food their children eat, especially when it is served at the grandmother’s house. Since polygamy is also practiced widely in such rural settings, it is not unexpected that children will eat in any of their step-mothers’ houses, a phenomenon which is culturally-accepted and valued. These findings confirm earlier studies which looked at food sharing between grandmothers and grandchildren as an effective way of creating and maintaining social relationships among people of different generations [19].

In the current study, children were fed based on the available foods. For example, about 70% of the mothers indicated that they fed their children on available foods, 20% fed their children based on their perceived feeding needs while 10% did not respond to the question. This showed a lack of knowledge on the feeding requirements of their children. Mothers who had attained higher levels of education had a higher likelihood of providing the best feeding management for their children. When asked to indicate which foods pre-school children should eat, majority (80%) of the mothers indicated that they did not know while 20% stated that they knew the right food. When the 20% were probed further, only 8% were able to report the correct foods that pre-school children require (i.e. largely protein foods) while 12% said they require carbohydrates. This was worrying since it is a pointer that some of the mothers in such rural settings had very little knowledge on the right food for pre-school children.

In terms of the order of serving food in the household, a low proportion of mothers served their husbands first during meal times. This is contrary to what is usually expected (i.e. in traditional Luo community, most women served their husbands as family heads prior to serving anyone else, probably to symbolize respect for the family head). However, some mothers felt that children should be served first. Reasons provided for serving the children first included the fact that the pre-school children needed more nutrients than anybody else within the family. Some of the respondents stated that: “*They feel hungry faster than the rest”.* This statement revealed that mothers were aware of the need to feed or give their children more attention or preference during meal time. It also indicated that the respondents had knowledge about the need to feed children constantly as they felt hungry faster than adults. Others correspondents provided other reasons by further stating that: “*The child is stubborn during meal times, so to avoid that, I have to give or serve him first”.* An additional scrutiny of the responses given by the mothers confirmed that although the children were served first, the food was not given on the basis of the nutritional needs of the child but availability. These findings suggest that the traditional norm of giving men priority in serving meals was quickly shifting to children.

## Conclusions

In conclusion, this study present data demonstrating that lower age, low education level and the mother’s getting engaged in business negatively affected their children’s feeding. In addition, it was demonstrated that majority of mothers within the age range of 15–19 years had most of their children feeding at their grandmothers’ houses. The order in which food was served in the household also affected the feeding of the children since if they were served first, there was a higher likelihood of the children being fed well. Based on these findings it is recommended that there is need for sensitization of the mothers on the management of feeding of children in Bondo County, Kenya. Through this, it is hoped that relevant interventions and management strategies would then be designed to target pre-school children particularly in such rural settings as in Bondo County, Kenya.

## Competing interests

There is no conflict of interest from any of the authors of the manuscript due to commercial or other affiliations.

## Authors’ contribution

ARW designed, carried out the survey studies in the field and participated in the drafting of the manuscript. CO, RNO, KO, and EOO designed the study and participated in the drafting of the manuscript. CO and SG performed the statistical analysis. All authors read and approved the final manuscript.

## Pre-publication history

The pre-publication history for this paper can be accessed here:

http://www.biomedcentral.com/1472-698X/13/47/prepub

## Supplementary Material

Additional file 1 **Questionaire for mothers of nursery school children.** The attached questionnaires were administered to mothers of nursery school children and used to collect information on factors in the management of feeding in nursery school children as perceived by their mothers in rural Bondo County, Kenya. Click here for file
