# Supplementary material for: Factors in the management of feeding in nursery school children as perceived by their mothers in rural Bondo County, Kenya
Source: BMC Int Health Hum Rights. 2013 Nov 15;13:47. doi: 10.1186/1472-698X-13-47 (PMC3842824; doi:10.1186/1472-698X-13-47)
Supplement: Additional file 1 — Questionaire for mothers of nursery school children. The attached questionnaires were administered to mothers of nursery school children and used to collect information on factors in the management of feeding in nursery school children as perceived by their mothers in rural Bondo County, Kenya. [file 1472-698X-13-47-S1.docx]

**QUESTIONAIRE FOR MOTHERS OF NURSERY SCHOOL CHILDREN**

Dear Parents,

We are very happy to visit you in your home today. The purpose of this visit is to ask you questions about the care you provide too your nursery school child in relation to food and health. In addition, we shall ask a few questions about your household.

The information that you provide will be kept confidential and will be used to make recommendations for improving health status of your children. Your name and that of your child will not be mentioned anywhere.

**QUESTIONNAIRE**

Household No._______________________________

Name of Mother (Optional) _____________________

Name of the Child (Optional) _____________________

Marital Status (Tick one)

1. Single 2. Married 3. Widowed

4. Divorced/separated.

**A. INFORMATION EDUCATION**

1. How many years of formal schooling did you complete? (Tick one)
2. None
3. 1-4
4. 5-8
5. 9 above
6. How many years of formal schooling did your husband complete? (Tick one)
7. None
8. 1-4
9. 5-8
10. 9 above
11. In which year were you born? _______________________
12. How old will you be at the end of this year? _________years.
13. In which year was your husband born? _________________

**B. HEALTH INFORMATION**

6. When your child (Name) is sick do you do? (Tick)

1. Take child to hospital /dispensary?

2. Give herbal treatment

3. Change the diet

4. Other (specify) ______________________________________

7. What food do you give to (name) when she/he is sick?

1. Porridge

2. Ugali and fish

3. Ugali and vegetables

4. Other (specify)

Why? ____________________________________________________

8. List four major common illnesses in this community.

Start with the most common

1._______________________

2._______________________

3._______________________

4.______________________

9. How does a sick child look like?

1. Weak and inactive

2. Feels cold and sleepy

3. Thin and does not eat

4. Other (Specify) ____________________________

10. With whom does your child (name) spend most of the time when not in school?

1. Mother

2. Other Children

3. Alone

4. Other (specify)____________________________

10. With whom does your child (name) spend most of the time when not in school?

1. Mother

2. Other Children

3. Alone

4. Other (specify) ___________________________

11. What activities is your child involved in when not in school?

1. Playing with other children

2. Washing utensils

3. Fetching firewood/water

4. Other (specify)

12. Where in this community do you go for health services when your child (name) is sick?

**C. DEMOGRAPHIC INFORMATION**

13. How many children of your own do you have a live together who are below 18 years of age?

1. 1-4
2. 5-10
3. 10-15
4. More than 15

14. How many daughters do you have who are below 18 years…………….daughters.

15. How many sons do you have who are below 18 years? --------------------------sons.

16. What position is your nursery school child in the family?………………………….

17. How many more children would you like to have in addition to those you have?...................................................................................................................................

18. Have you discussed this with your husband?

1. Yes 2. No

19 Which meals does your child (name) usually eat at home?

1. Breakfast 2. Lunch 3. Supper
2. Other (specify)------------------------------------------------

20. What does your child (name) usually eat for breakfast?

1. Porridge 2. Tea and Bread

3. Ugali and Fish 4. Other (Specify)

21. What does your child (name) usually eat for lunch?

1. Ugali ad Fish

2. Ugali and Vegetables

3. Beans mixed with Maize (Nyoyo)

4. Other (Specify)

22. What does your child (name) usually eat for supper?

1. Ugali and Fish

2. Ugali and Vegetables

3. Beans mixed with maize

4. Other (Specify)

23. Who usually cooks meals in this house?

1. Mother

2. Older children

3. Maid

4. Other (specify)

24. With whom does your child (name) usually eat?

1. Mother

2. Father

3. Other children

4. Other (specify)

25. In this household who eats first at the main meal?

1. Father

2. Children

3. Mother

4. Eat together

Why………………………………………………………………………………………

26. Where else does your child (name) eat?

1. At neighbor’s house

2. At stepmothers’ house

3. Grandmothers house

4. Other (specify)

**D. INFORMATION ON ECONOMICS STATUS**

27. What work do you do to earn a living?

1. Salaried employment

2. Farmers

3. Business

4. Other (specify)

28. What work does your husband do to earn a living?

1. Salaried employment

2. Farmers

3. Business

4. Other (specify)

29 What work do your children help with at the household?

1. Cooking
2. Fetching water
3. Washing utensils
4. Other (specify)

30. How many cows does your husband have? -------------------------------------cows.

31. How many goats does your husband have?................................goats

32. What other animals do you have?....................................................................................

33. How many hectares of land does your husband have for food crop cultivation?...................hectares.

34. Does your husband own a bicycle?

1. No 2. Yes

35 Does your husband own a vehicle?

1. No 2. Yes

36. Type of house for respondent?

1. Temporary grass thatched

2. Semi-permanent

3. Permanent

37. Does your husband own a working radio?

1. Yes 2. No

**E. INFORMATION ON MARITAL UNION**

38. How many other wives does your husband have?..........................wives

39. What position are you?...............position

40. Are there taboos that are related to food that you give to your nursery school child?

1. No
2. Yes
3. Don’t know

41. If Yes, Explain………………………………………………………………….

42. Do you think there is need to set up a food programme for nursery school children in this community?

1. Yes 2. No

43. Why?............................................................................................................

44. Do you think a training programme on child nutrition would be beneficial for nursery school mothers in this community?

1. Yes 2. No

45. Why?................................................................................................................................

**Thank you for finding time to answer my questions.**
